# Assessing the microbiota of the snail intermediate host of trematodes, *Galba truncatula*

**DOI:** 10.1186/s13071-024-06118-7

**Published:** 2024-01-23

**Authors:** Peter McCann, Christopher McFarland, Julianne Megaw, Karen Siu-Ting, Cinzia Cantacessi, Gabriel Rinaldi, Geoffrey N. Gobert

**Affiliations:** 1 School of Biological Sciences, Queen’s University Belfast, Belfast, UK; 2 Department of Veterinary Medicine, University of Cambridge, Cambridge, UK; 3 Department of Life Sciences, University of Aberystwyth, Aberystwyth, UK

**Keywords:** Microbiota, 16S-sequencing, Microbiome, Snail, Parasite, Host-Parasite interactions, *Galba truncatula*

## Abstract

**Background:**

The microbiome is known to play key roles in health and disease, including host susceptibility to parasite infections. The freshwater snail *Galba truncatula* is the intermediate host for many trematode species, including the liver and rumen flukes *Fasciola hepatica* and *Calicophoron daubneyi*, respectively. The snail-parasite system has previously been investigated. However, the specific interaction between the snail-associated microbiota and intra-snail developmental stages of trematodes has yet to be explored.

**Methods:**

*Galba truncatula* snails were collected from farms in Northern Ireland and trematode infection was diagnosed using PCR. High-throughput sequencing analysis of the bacterial 16S ribosomal DNA V3-V4 hypervariable regions was subsequently applied to characterise the microbiota of both uninfected and infected snails.

**Results:**

We first showed that the snail harboured microbiota that was distinct for its environment. The microbiota of infected snails was found to differ significantly from that of uninfected snails. In particular, the bacterial genera *Mycoplasma* and *Methylotenera* were significantly more abundant in infected snails, while genera *Sphingomonas* and *Nocardioides* were predominantly associated with uninfected snails.

**Conclusion:**

These findings pave the way to future studies on the functional roles of bacteria in host-parasite relationships.

**Graphical Abstract:**

**Figure:**
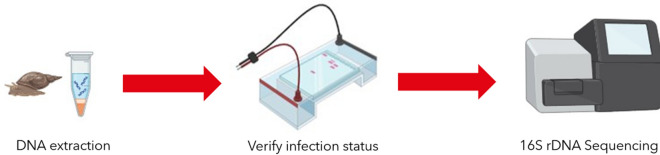

**Supplementary Information:**

The online version contains supplementary material available at 10.1186/s13071-024-06118-7.

## Background

Freshwater snails, such as *Galba truncatula*, act as intermediate hosts for many trematode parasites, including the liver fluke *Fasciola hepatica* and the rumen fluke *Calicophoron daubney*i [1]. *Fasciola hepatica*, the causative agent of fascioliasis, is a zoonotic parasite that infects a wide range of hosts, including humans and livestock. A recent review suggests that *F. hepatica* infects livestock globally, except in Antarctica, and is thought to infect 50 million people [2]. Humans are most commonly infected following ingestion of aquatic vegetation, water and/or vegetables contaminated with parasite metacercariae [3]. Recently, there has been growing interest in the rumen fluke *C. daubneyi* in UK agriculture due to its apparent increase in distribution and use of *G. truncatula* as an intermediate host [4].

The flukes *F. hepatica* and *C. daubneyi* exhibit a complex life-cycle with free-living and in-host life stages. The adult worms dwell in the definitive mammalian host and shed eggs that are passed in host faeces. The eggs embryonate in fresh water and develop into miracidia that after hatching seek out and infect the intermediate host snail. Upon reaching a suitable intermediate host, miracidia penetrate the snail and undergo asexual clonal reproduction with metamorphic transformations through sporocyst, rediae and cercariae phases [5]. The life-cycle is completed when free-swimming cercariae leave the intermediate host and encyst upon vegetation as infective metacercariae.

While the microbiota is defined as “a characteristic microbial community occupying a reasonable well-defined habitat which has distinct physio-chemical properties” [6], the term microbiome refers to the pool of genomes from all of the microorganisms comprising the microbiota [6]. The host microbiome is known to be intricately linked to immunity and to play a role in host–pathogen interactions [7, 8]. Whereas most microbiome studies of trematodiases have mainly focused on the mammalian host, studies on the microbiota of the mollusc intermediate host are scarce [9–12]. In the definitive host, it is recognised that the parasitic infection can directly interact with the gut microbiota [13–15]; however, despite the importance of intermediate snail hosts in the transmission of trematode diseases, the relationships between helminths and the snail-associated microbiota are yet to be thoroughly investigated.

The aim of the current study was to profile the microbiota of *G. truncatula* snails collected from local farms in Northern Ireland. The presence of trematode species was confirmed by PCR in 12 of the 56 snails collected. Subsequent high-throughput sequencing analysis of the bacterial 16S ribosomal DNA (rDNA) hypervariable regions V3 and V4 characterised the microbiota of uninfected and infected snails and identified significant differences in the composition of the microbiota relative to infection status.

## Methods

### Snail and soil sample collection

Snails of unknown infection status (*n* = 56) were collected from two farms located in Northern Ireland (NI) in September 2021. Farm 1 was a sheep farm located in County Antrim, NI with approximately 200 sheep; Farm 2 had approximately 50 cattle and was located in County Tyrone. The two farms were selected based on previous reports by the Queen’s University of Belfast confirming liver and rumen fluke infections by faecal egg counts using published protocols [16]. Snail sampling locations on the farms were selected based on an assessment of suitable snail habitats, including the presence of bare mud and proximity of known bioindicator plant species [17]. Previous studies on *G. truncatula* habitat use have confirmed that open drainage furrows, drainage ditches and rush beds surrounding natural springs are the preferential site for snail populations [18].

The habitat structure of each farm collection area is shown in Additional file 1: Figure S1 and Additional file 2: Figure S2. Livestock retained free access to sample collection sites during the months prior to sample collection.

Snails were collected at five collection points across the sampling location on each farm along the edge of a water body within the boundaries of a 0.25-m^2^ quadrat during a 10-min search [4]. A 10-min search within the bounds of each quadrat was selected to ensure that the snail searching effort within each quadrat was standardised across the sampling location. All snail identifications and collections were completed by the same researcher. Snails were collected using sterile tweezers and transferred into individual sterile collection tubes for transport to the laboratory at 4 °C. To examine the microbial community of the surrounding snail habitat, soil samples were also collected within the bounds of the 0.25-m^2^ quadrat where snails were collected. At each quadrat collection point, three individual tubes were filled with approximately 25 g of soil that was flaked off the surface (maximum depth: 2 cm) using a spatula. This equated to a total of 15 individual tubes of soil across the entire sampling location. Soil samples were collected from quadrats that showed the presence of snails. Individual tubes of soil from each of the five collection points (i.e. 1 tube per quadrat collection point) were pooled to generate three soil samples, each consisting of five original collections. This was completed three times to account for the three individual tubes of soil collected within each quadrat, generating a total of three larger soil pools made up of all 15 collected tubes of soil with five original biological replicates in each larger pool. These samples were considered to encompass the diversity of the soil microbiota the snails have the potential to be exposed to during their life at the water body. Soil samples were stored at − 20 °C prior to DNA extraction.

### Snail DNA extraction protocol

Snail specimens were stored at − 20 °C prior to genomic DNA (gDNA) extraction. Once thawed, the shell of each individual snail was disinfected 3 times with 70% ethanol following protocols outlined in [19], to prevent the introduction of exogenous bacterial contamination, and subsequently removed using forceps under a dissection light microscope. Once the shell had been removed, each snail was individually transferred into a 2-ml microcentrifuge tube, and each tube containing a single snail was then snap frozen in liquid nitrogen before the addition of a single 5-mm stainless steel bead (Qiagen, Hilden, Germany). Snail tissue was homogenised by bead beating on the Qiagen TissueLyser LT bead mill at 50 Hz for 3 min. DNA was extracted using the DNeasy® Blood and Tissue kit (Qiagen) with a 3-h Proteinase K incubation at 56 °C. All samples were processed using a single DNA extraction kit, with extractions completed by the same researcher.

Blank DNA extraction controls (kitomes) consisting of 50 µl DEPC-treated water were included during extractions to monitor the presence of contaminating DNA in the extraction reagents. ZymoBIOMICS Microbial Community Standards (MCS) (Zymo Research, Irvine, CA, USA) consisting of a defined species composition of *Listeria monocytogenes* (12%), *Pseudomonas aeruginosa* (12%), *Bacillus subtilis* (12%), *Escherichia coli* (12%), *Salmonella enterica* (12%), *Lactobacillus fermentum* (12%), *Enterococcus faecalis* (12%), *Staphylococcus aureus* (12%), *Saccharomyces cerevisiae* (2%) and *Cryptococcus neoformans* (2%) were extracted using the same DNeasy® Blood and Tissue Kit (Qiagen) to validate the extraction procedure. DNA was eluted in 50 µl of elution buffer AE. Kitomes and mock community extractions were completed in parallel with sample processing to monitor potential contamination during the procedure.

### Soil DNA extraction protocol

The three larger pooled soil samples were thawed, and the total mass of each sample measured; stones, large plant debris or large invertebrates were removed using sterile tweezers before manual homogenisation using a sterile spatula. The sample was then placed in a spice blender with a removable plastic compartment and mechanically homogenised using twenty 1-s pulses. The removable plastic compartment of the blender was sterilised by first removing large particulate matter followed by submersion in 50% bleach for 30 min, and then rinsed in DEPC-treated water. The sample was then further mixed using a spatula, and three 0.25-g aliquots (i.e. technical replicates) were removed from the pooled sample and added to a PowerBead Pro tube (Qiagen). DNA extraction was completed using the DNeasy® PowerSoil® Pro Kit (Qiagen) according to the manufacturer’s guidelines. Homogenisation steps were completed on a TissueLyser II biological sample disrupter (Qiagen) according to the manufacturer's recommended settings. The final DNA elution step consisted of adding 100 µl of solution C6 (Qiagen) into each of the three larger pooled collections, resulting in a total of nine DNA extractions of 0.25 g of soil each. The eluted DNA from three DNA extractions (equating to 0.75 g of total soil) from each of three larger soil pools was pooled together in equal proportions, resulting in a total of six pooled aliquots of DNA for sequencing, i.e. three aliquots for each study farm.

### Confirmation of snail infection status

The snail infection status was confirmed by conventional PCR (cPCR). Previously reported pan-trematode primers targeting the rDNA internal transcribed spacer 2 (ITS2) gene locus (forward: 5’–TGTGTCGATGAAGAGCGCAG–3’; reverse: 5’–TGGTTAGTTTCTTTTCCTCCGC–3’) were used [20], and the amplicon was then sent for Sanger sequencing to determine the trematode species present. The Taq PCR Mastermix Kit (Qiagen) was used and consisted of 2 µl of template DNA added to the 25-µl reaction mix (13 µl of Taq, 8 µl of DEPC-treated water, 1 µl of the forward and reverse primers normalised to 100 µM). The thermocycler was programmed to 95 °C for 10 min; followed by 35 cycles of 95 °C for 15 s, 56 °C for 15 s and 72 °C for 30 s; with a final extension of 10 min at 72 °C. A gel electropherogram was used to identify positive samples with an expected band size of 428—500 bp. Coinfections were identified by PCR using pan-trematode primers followed by Sanger sequencing of each band present in the gel (Additional file 3: Figure S3). Species-specific primers were also used for *F. hepatica* and *C. daubneyi * (forward: 5’–TGTGTCGATGAAGAGCGCAG–3’; reverse: 5’–TGGTTAGTTTCTTTTCCTCCGC–3’) using the same conditions as described above. The band was then excised from the gel, purified using the Qiagen Gel Clean-Up Kit (Qiagen) and sequenced by Eurofins TubeSeq service (Eurofins Scientific, Luxembourg City, Luxembourg). DNA purity was determined using a Denovix Nanodrop spectrophotometer (Thermo Fisher Scientific) and quantified using a Qubit fluorometer (Thermo Fisher Scientific) using the high-sensitivity kit. The parasite species were identified using NCBI BLAST, with a nucleotide BLAST being used against all databases, using default parameters. The cumulative read counts for each sample stratified by farm is shown in Additional file 4: Figure S4.

### Microbiota sequencing of snails and soil

To examine the microbiota of trematode-uninfected/-infected snails and the tentative microbial community of the surrounding soil environment, we carried out 16S rRNA sequencing on a total of 44 DNA samples, including samples from 18 uninfected snails (selected based on having the highest DNA concentration and purity determined via spectrometry on the Nanodrop spectrophotometer; Thermo Fisher Scientific), 12 trematode-infected snails, six soil DNA aliquots, four negative controls (“kitomes”) and four mock community DNA extractions. The remaining 26 uninfected snails were not analysed. Sequencing was carried out by Randox Clinical Laboratory Services (Antrim, Northern Ireland), with individual 16S rRNA amplicon libraries generated using proprietary primers targeting the V3-V4 variable region. Paired-end sequencing with 300-bp read length was performed on an Illumina MiSeq sequencer (Illumina Inc., San Diego, CA, USA; provided by Genome Québec) using Illumina Miseq Reagent V3 chemistry (2 × 300 bp) according to the manufacturers protocol. Sample concentration was normalised prior to sequencing, and equal volumes of each sample were pooled. The samples were loaded at 1 nM concentration and passed both Illumina's PassFilter % and Q 30% metrics for a 2 × 300-bp sequencing run on the Miseq sequencer. The sample reads were in the Cassava 18 paired end demultiplexed format.

### 16S rRNA microbiota analysis

The FASTQ files were analysed with the software package Quantitative Insights in Microbial Ecology 2 (QIIME2) [21] using the DADA2 plugin [22]. Reads were truncated at left-13 and len-250 based on the read quality from FastQC v0.11.9 [23]. Sequences were denoised, and feature data were used to generate feature tables. The scripts and commands used from the QIIME2 pipeline as well as other software can be found in the open access Github repository (https://github.com/agalychnica/McCann_etal_Snail_microbiota). After denoising and generating an amplicon sequence variant (ASV) count table, a classify-consensus sklearn function was used to assign taxonomy using the Naïve Bayes classifier trained on a Silva 132_99_V3V4 database (downloaded from https://github.com/Jiung-Wen/q2-silva-V3V4classifier) that matches our 16S target regions. Sample metadata used for the analysis can be found in Additional file 8: Table S1. The resulting taxonomy data from the sklearn classifier was integrated into the ASV count table (Additional file 9: Table S2). Reads present in our negative control “kitome” samples were accounted for using the Decontam R package [24] and taxa found to be putative contaminants (threshold: 0.5) using the prevalence method in this analysis (13 taxa/ASVs in total) were removed using shell scripting (all scripts supplied in https://github.com/agalychnica/McCann_etal_Snail_microbiota). The table filtered from putative contaminants was then used for further downstream statistical analyses in the online tool MicrobiomeAnalyst [25]. Low abundance (total ASV reads < 4) or low variance (ASVs present in < 20% of the samples) taxa were excluded to account for sequencer error. With these filters applied, a total of 997 low-abundance ASVs were removed based on prevalence and 39 low-variance ASVs were removed based on interquartile range. The number of ASVs remaining after data filtering was 348. The data were rarefied when kitomes and community standards were excluded from the diversity and LEfSe analysis. The data were not transformed, but total sum scaling was applied. Data from two samples (28Jan22-EXT03, 28Jan22-EXT09) were excluded based on observations of the alpha rarefaction (Additional file 5: Figure S5), suggesting low read depth compared to the rest of the samples included in the analysis, possibly due to sequencing errors. Sequencing reads have been uploaded to the NCBI Sequencing Read Archive (PRJNA992680).

Alpha diversity was calculated in MicrobiomeAnalyst using metrics that included Chao1, observed richness, Shannon diversity index, Simpson diversity index and Fisher’s diversity index. Differences between infected and uninfected snails were then tested using the Mann-Whitney U statistical test for each diversity index calculated at the genus level. Beta diversity was calculated using the Bray–Curtis dissimilarity, Jensen-Shannon and Jaccard diversity metrics via permutational multivariate analysis of variance (PERMANOVA) at the genus level. Linear discriminant analysis (LDA) Effect Size (LEfSe) [25] was used to determine the association between metadata variables and beta diversity at taxonomic levels from phylum to genus.

## Results

### Host snail microbiota in trematode-infected snails

**Fig. 1:**
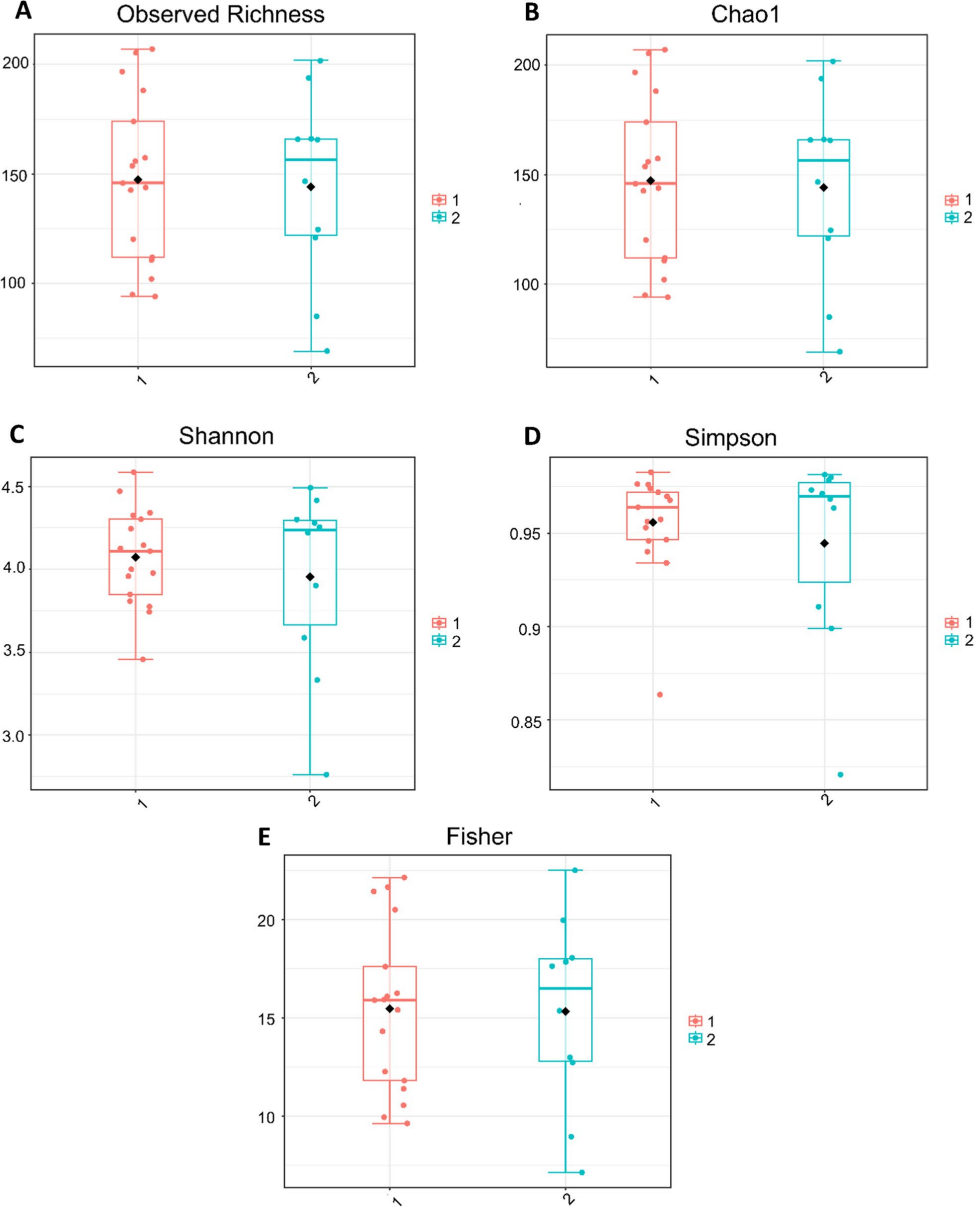
Alpha diversity of *Galba truncatula* snail samples stratified by farm, presented at the genus level. **a** Comparison of observed richness revealing no significant differences in alpha diversity between snails collected from each farm (*P*-value = 0.98; Mann–Whitney *U* statistic = 84). **b** Comparison of Chao1 richness revealing no significant differences in alpha diversity between snails collected from each farm (*P*-value = 0.98; Mann–Whitney *U* statistic = 84). **c** Comparison of Shannon’s diversity index revealing no significant differences in alpha diversity between snails collected from each farm (*P*-value = 0.98; Mann–Whitney *U* statistic = 86). **d** Comparison of Simpson’s diversity index revealing no significant differences in alpha diversity between snails collected from each farm (*P*-value = 0.71; Mann–Whitney *U* statistic = 77). **e** Comparison of Fisher diversity index revealing no significant differences in alpha diversity between snails collected from each farm (*P*-value = 0.90; Mann–Whitney *U* statistic = 82). The numbers 1 and 2 to right of boxplots refer to Farm 1 (sheep farm located in County Antrim, in orange) and Farm 2 (with approximately 50 cattle, located in County Tyrone, in blue), respectively

**Fig. 2:**
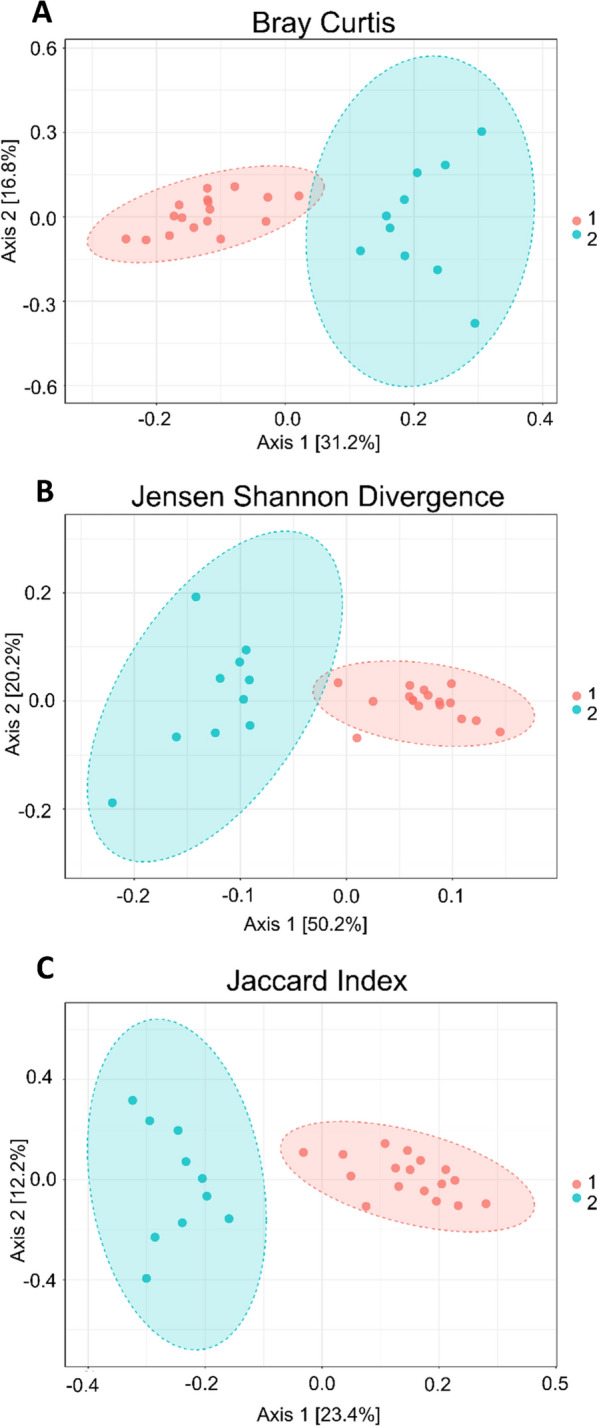
Beta diversity of *Galba truncatula* snails collected from each farm, presented at the genus level. **a** Beta diversity compared using Bray–Curtis dissimilarity (PCoA) distances revealing significant differences in the diversity of the microbiota of snails collected from each farm (PERMANOVA:* F*-value = 9.55;* R*^2^ = 0.28; *P*-value = 0.001). **b** Beta diversity compared using Jensen-Shannon divergence (PCoA) distances, revealing significant differences in the diversity of the microbiota of snails collected from each farm (PERMANOVA:* F*-value = 19.32;* R*^2^ = 0.44; *P*-value = 0.001). **c** Beta diversity compared using Jaccard index (PCoA) distances, revealing significant differences in the diversity of the microbiota of snails collected from each farm (PERMANOVA:* F*-value = 6.76;* R*^2^ = 0.21;* P*-value = 0.001). The numbers 1 and 2 to right of boxplots refer to Farm 1 (sheep farm located in County Antrim, in orange) and Farm 2 (with approximately 50 cattle, located in County Tyrone, in blue), respectively. PERMANOVA, Permutational multivariate analysis of variance; PCoA, principal co-ordinates analysis

**Fig. 3:**
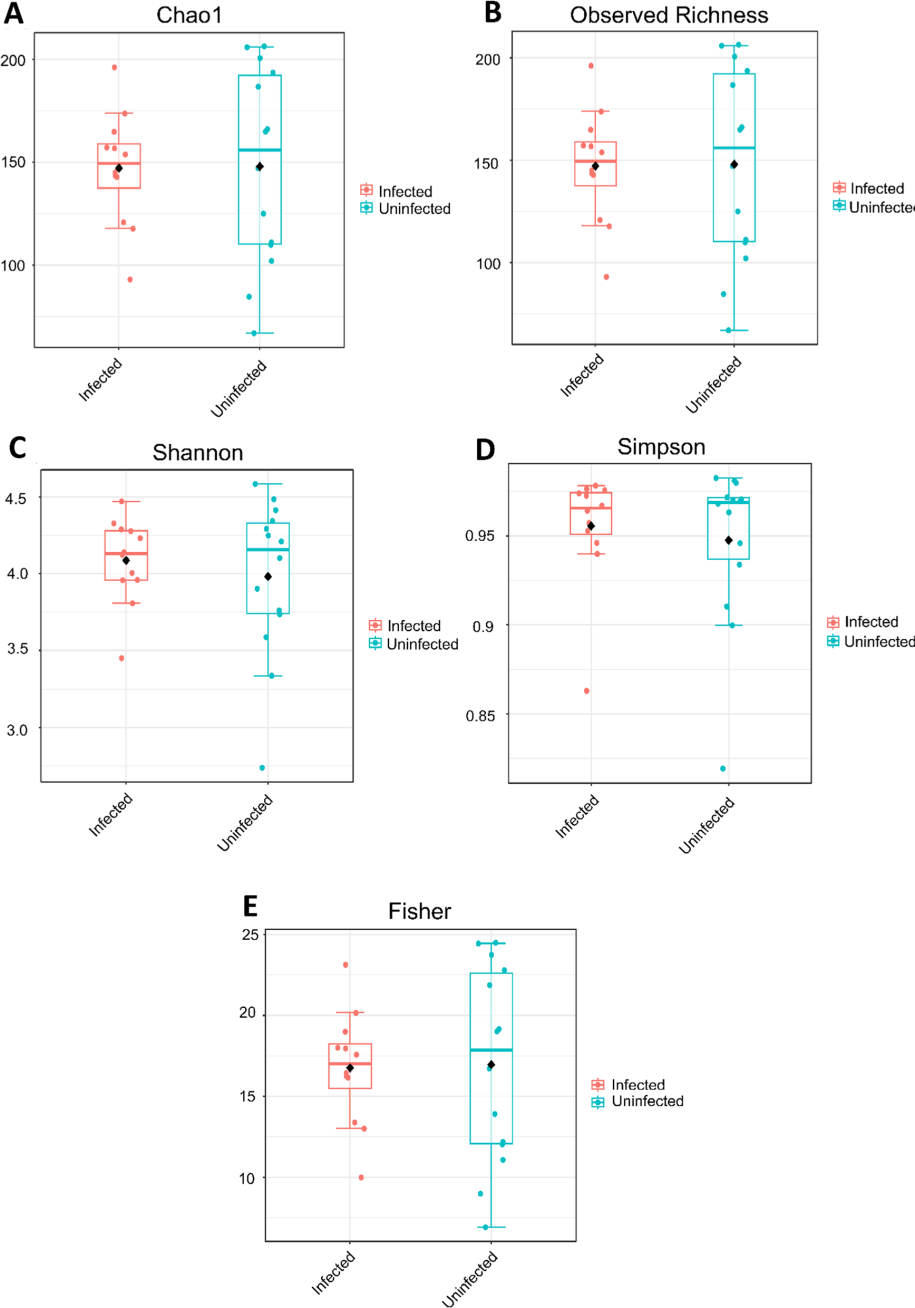
Alpha diversity of infected and uninfected *Galba truncatula* snail samples, presented at the genus level. **a** Comparison of Chao1 richness, revealing no significant differences in alpha diversity between infected and uninfected snails (*P*-value = 0.79; Mann–Whitney *U* statistic = 78.5). **b** Comparison of observed richness, revealing no significant differences in alpha diversity between infected and uninfected snails (*P*-value = 0.79; Mann–Whitney *U* statistic = 78.5). **c** Comparison of Shannon’s diversity index, revealing no significant differences in alpha diversity between infected and uninfected snails (*P*-value = 0.89; Mann–Whitney *U* statistic = 87). **d** Comparison of Simpson’s diversity index, revealing no significant differences in alpha diversity between infected and uninfected snails (*P*-value = 0.87; Mann–Whitney *U* statistic = 94). **e** Comparison of Fisher diversity index, revealing no significant differences in alpha diversity between infected and uninfected snails (*P*-value = 0.80; Mann–Whitney *U* statistic = 78.5)

**Fig. 4:**
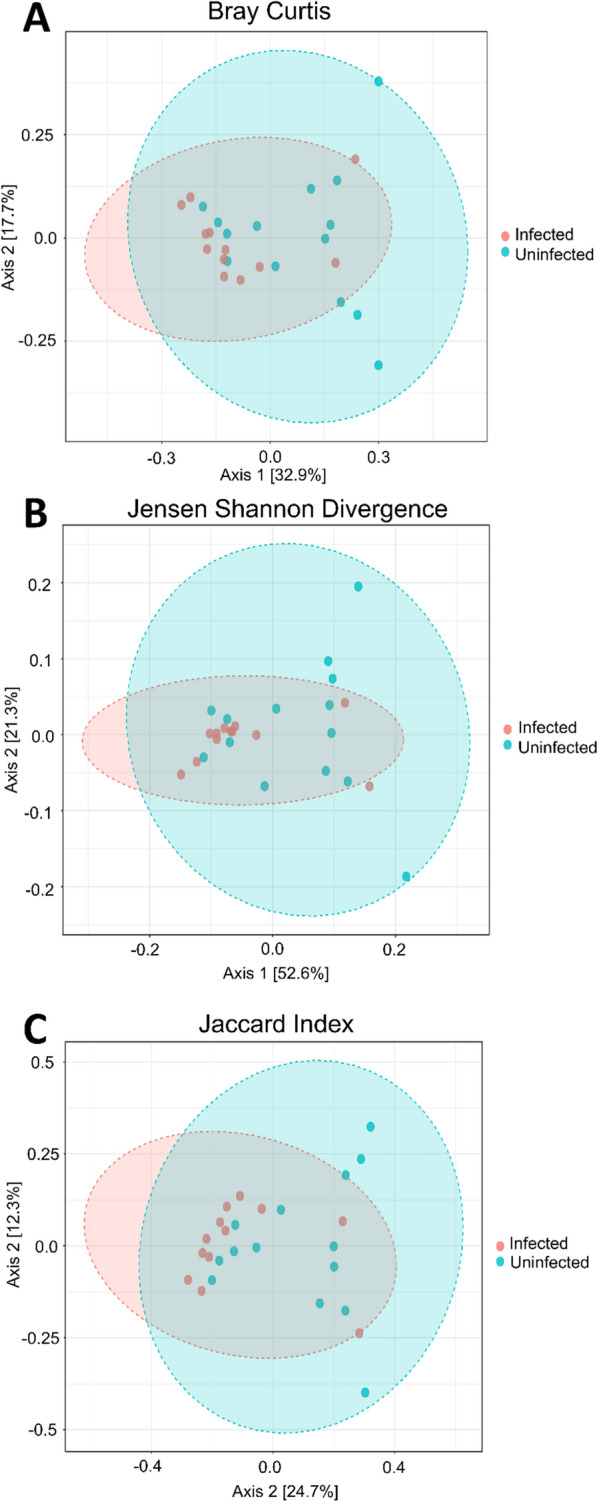
Beta diversity of infected and uninfected *Galba truncatula* snails, presented at the genus level. **a** Beta diversity compared using Bray–Curtis dissimilarity (PCoA) distances, revealing significant differences in the diversity of the microbiota of infected and uninfected snails (PERMANOVA:* F*-value = 2.33;* R*^2^ = 0.09; *P*-value = 0.017). **b** Beta diversity compared using Jensen-Shannon divergence (PCoA) distances, revealing significant differences in the diversity of the microbiota of infected and uninfected snails (PERMANOVA:* F*-value = 2.99;* R*^2^ = 0.11; *P*-value = 0.028). **c** Beta diversity compared using Jaccard index (PCoA) distances, revealing significant differences in the diversity of the microbiota of infected and uninfected snails (PERMANOVA:* F*-value = 2.07;* R*^2^ = 0.08; *P*-value = 0.014). PERMANOVA, Permutational multivariate analysis of variance; PCoA, principal co-ordinates analysis

A total of 46 samples were included in the post-sequencing analysis with a range of 1146–216,898 reads per sample, and an average of 104,707 reads. A summary for each sample is shown in Additional file 6: Figure S4 and Additional file 6: Figure S6. The resulting samples were stratified in the metadata by farm ID and infection status (uninfected/infected). Although different trematodes species were identified, the small number of snails representing single species-specific infections limited further specific microbiota analysis. Firstly, we examined differences in alpha and beta diversity depending on the farm the snails were collected from, and found that there were no significant differences in alpha diversity (Fig. 1), but significant differences were observed in their beta diversity metrics (Fig. 2). Differences in microbial diversity depending on whether the snail was infected or uninfected were analysed. We found that there were no significant differences in alpha diversity (Fig. 3) between infected and uninfected snails, but there were significant differences in beta diversity (Fig. 4) between the two groups based on the PERMANOVA analysis. No significant differences in beta diversity were noted when comparing the trematode species present. Of the 12 snails representing our infected group, we found one case of *C. daubneyi* (OQ102036.1), [26], one case of *Neoglyphe sobolevi* (MK294328.1) and five cases of coinfection with *Rubenstrema exasperatum* (OQ354216.1, OQ354215.1) and *N. sobolevi* (MK294328.1, MK294329.1) The remaining five samples were positive for the pan-trematode PCR but did not return a hit in the NCBI database with a threshold set at 99%. Remarkably, we did not find any *F. hepatica* infections, possible due to regular anthelmintic treatment on the farms.

**Fig. 5:**
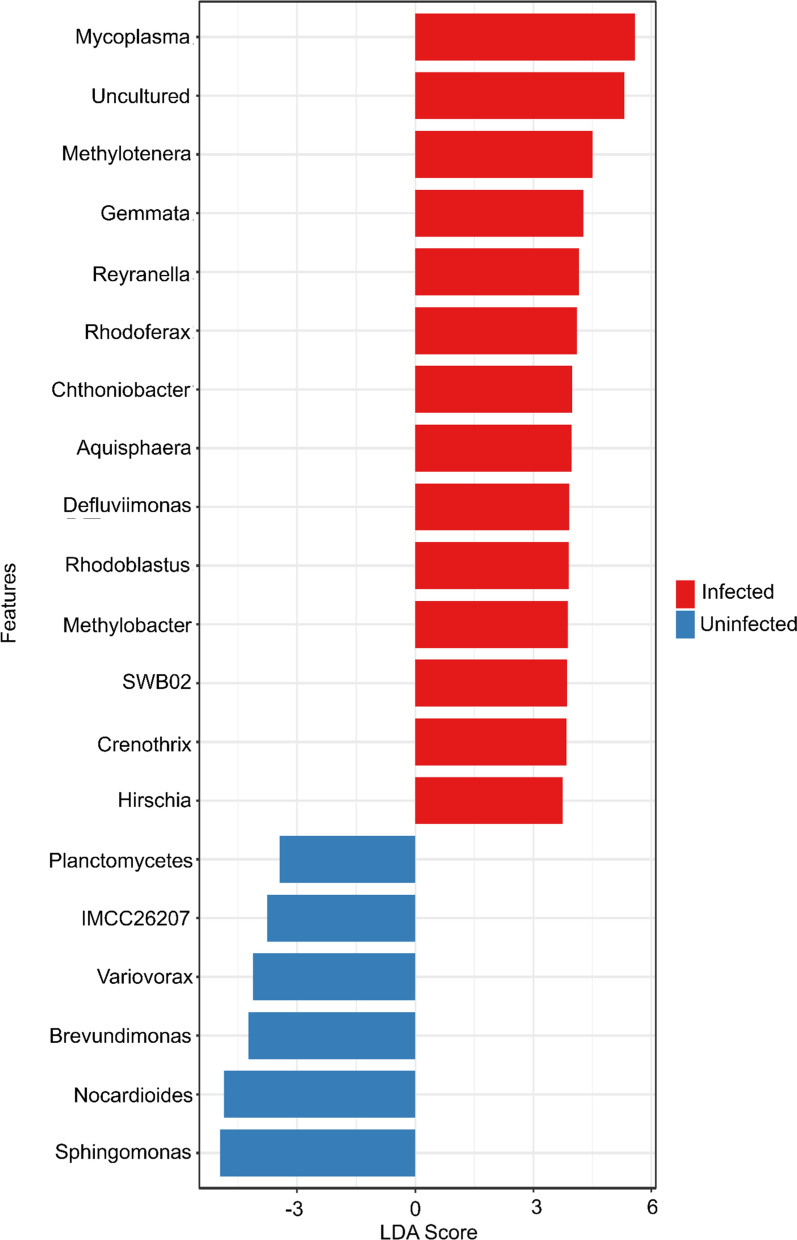
LEfSe analysis of snails according to infection status. The genera of bacteria found in higher frequency in infected snails are shown as red bars on the top right of the graph. Those genera found in greater frequency in naïve snails are shown as blue bars on the bottom left of the graph. LEfSe, Linear discriminant analysis Effect Size

LEfSe was performed to determine the bacterial genera which were significantly different when comparing beta diversity of snail microbiota in infected versus uninfected snails (Fig. 5). At the phylum level, we found that Mycoplasma (LDA = 5.58), Bacteroidetes (LDA = 5.14) and Planctomycetes (LDA = 5.08) had a significantly higher abundance in infected snails, whereas Firmicutes (LDA = − 5.36), Cyanobacteria (LDA = − 5.35) and Actinobacteria (LDA = − 5.20) were significantly more abundant in uninfected snails (Table 1). The genera *Mycoplasma* (LDA = 5.58), *Methylotenera* (LDA = 4.51) and *Gemmata* (LDA = 4.27) were the most highly associated with infected snails, while the genera *Sphingomonas* (LDA = − 4.95), *Nocardioides* (LDA = − 4.86) and *Brevundimonas* (LDA = − 4.23) were more associated with naïve snails. The complete list of taxa identified by LEfSe linked to the presence or absence of helminth infection is given Additional file 10: Table S3, stratified by phylum, class, order, family and genus.

**Table 1:** Results of linear discriminant analysis Effect Size analysis presented at the phylum level stratified by infection status

Phylum	*P*-values	False discovery rate	Infected snails	Uninfected snails	LDA score^a^
D_1__Tenericutes	0.024357	0.14308	1,171,000	416,270	5.58
D_1__Bacteroidetes	0.000906	0.016316	486,080	210,850	5.14
D_1__Planctomycetes	0.031795	0.14308	868,550	627,050	5.08
D_1__Verrucomicrobia	0.52586	0.80844	382,380	339,130	4.34
D_1__Gemmatimonadetes	0.047757	0.14327	58,121	35,108	4.06
D_1__WPS_2	0.018436	0.14308	13,692	786.67	3.81
D_1__Dependentiae	1	1	21,842	12,599	3.66
D_1__Patescibacteria	0.67827	0.81393	55,722	48,374	3.57
Not Assigned	0.60525	0.80844	12,619	9755.9	3.16
D_1__Armatimonadetes	0.62879	0.80844	4201.5	2258.7	2.99
D_1__Nitrospirae	0.54654	0.80844	9103.3	7580.6	2.88
D_1__Acidobacteria	0.7697	0.81497	297,760	299,580	− 2.96
D_1__Chloroflexi	0.4908	0.80844	29,083	32,444	− 3.23
D_1__Chlamydiae	0.49452	0.80844	77,855	82,046	− 3.32
D_1__Proteobacteria	0.49452	0.80844	5,329,500	5,477,000	− 4.87
D_1__Actinobacteria	0.040393	0.14327	548,520	866,780	− 5.2
D_1__Cyanobacteria	0.73268	0.81497	271,180	716,620	− 5.35
D_1__Firmicutes	0.078983	0.2031	362,800	815,790	− 5.36

^a^Positive least discriminant analysis (LDA) scores indicate phyla which were significantly more abundant in infected snails compared to uninfected snails. Negative LDA scores indicate phyla which were significantly more abundant in uninfected snails compared to infected cells

### Evident differences between the environmental and snail microbiomes

**Fig. 6:**
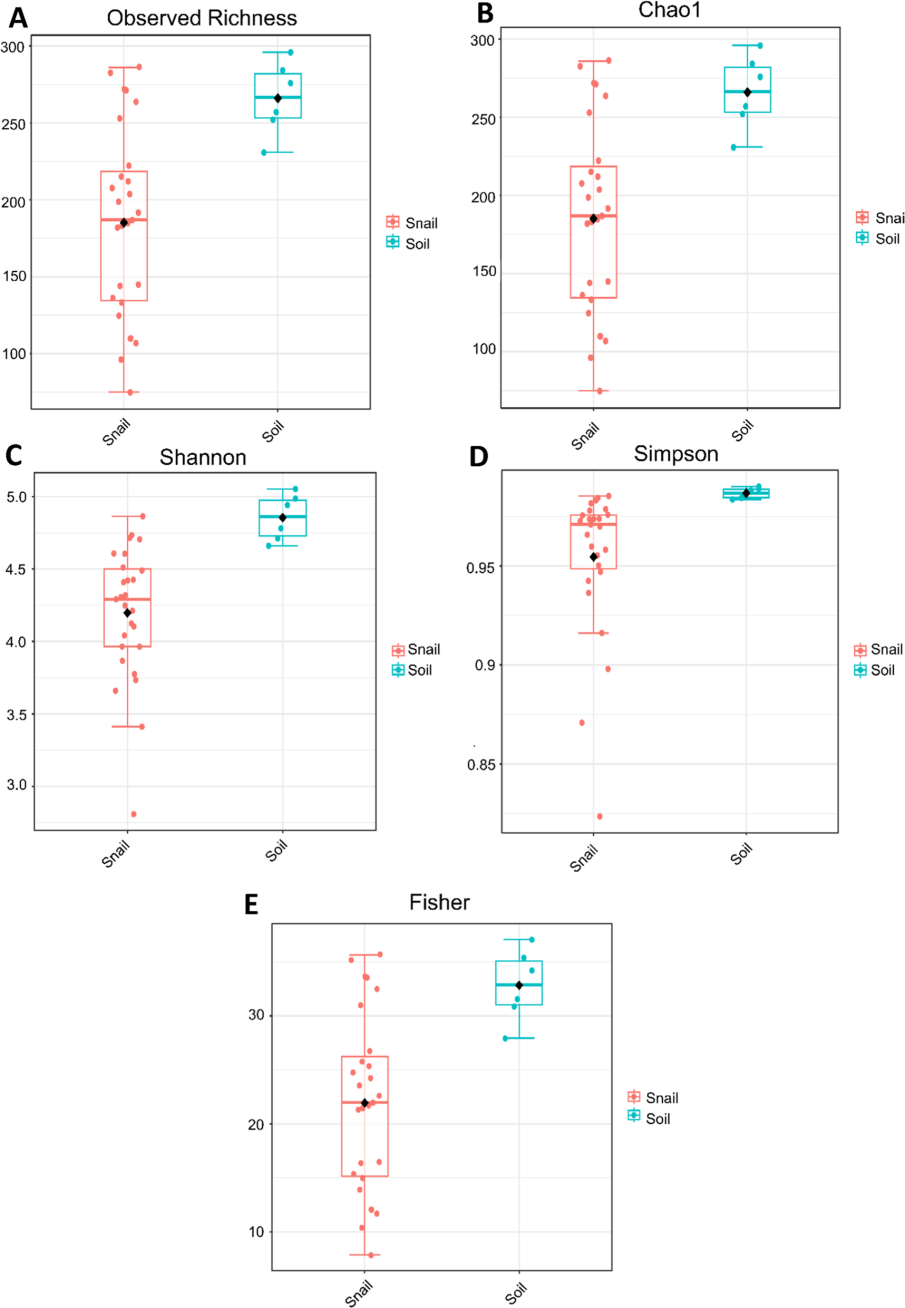
Alpha diversity. Diversity of *Galba truncatula* snail samples and environmental soil samples collected from each farm, presented at the genus level. **a** Comparison of observed richness, revealing significant differences in alpha diversity between the snails and soil (*P*-value = 0.004; Mann–Whitney* U* statistic = 20). **b** Comparison of Chao1 richness, revealing significant differences in alpha diversity between the snails and soil (*P*-value = 0.004; Mann–Whitney* U* statistic = 20). **c** Comparison of Shannon’s diversity index, revealing significant differences in alpha diversity between the snails and soil (*P*-value = 0.0001; Mann–Whitney* U* statistic = 8). **d** Comparison of Simpson’s diversity index, revealing significant differences in alpha diversity between the snails and soil (*P*-value = 3.4309e-05; Mann–Whitney* U* statistic = 5). **e** Comparison of Fisher’s diversity index, revealing significant differences in alpha diversity between the snails and soil (*P*-value = 0.004; Mann–Whitney* U* statistic = 20)

**Fig. 7:**
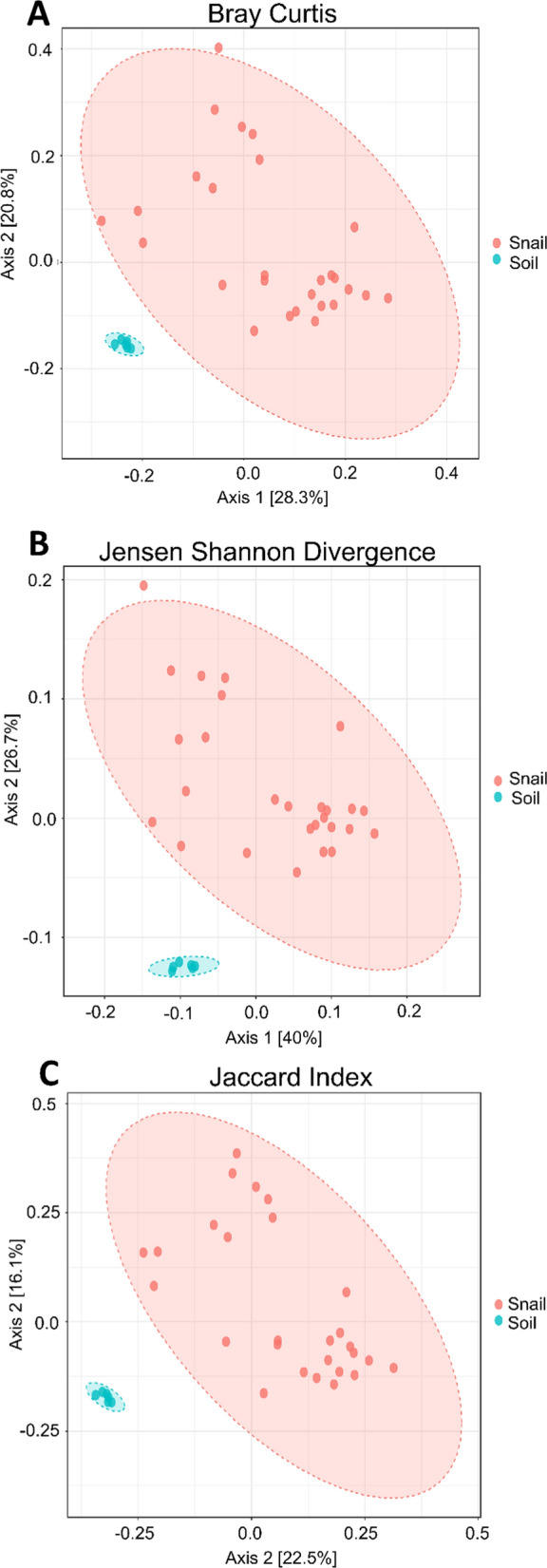
Beta diversity of *Galba truncatula* snail samples and environmental soil samples collected from each farm, presented at the Genus level. **a** Beta diversity compared using Bray-Curtis dissimilarity (PCoA) distances revealing significant differences in the diversity of the microbiota of *G. truncatula* and environmental soil ([PERMANOVA] *F*-value: 7.37; R-squared: 0.19; *p*-value: 0.001). **b** Beta diversity compared using Jensen–Shannon divergence (PCoA) distances revealing significant differences in the diversity of the microbiota of *G. truncatula *and environmental soil ([PERMANOVA] *F*-value: 9.88; *R*-squared: 0.24; *p*-value: 0.001). **c** Beta diversity compared using Jaccard index (PCoA) distances revealing significant differences in the diversity of the microbiota of *G. truncatula* and environmental soil ([PERMANOVA] *F*-value: 6.52;* R*-squared: 0.17; *p*-value: 0.001)

When comparing the differences in alpha diversity between the snails and soil replicates (Fig. 6), we found that there were significant differences in all diversity metrics, with higher diversity in the soil replicates. We also found significant differences in all beta diversity metrics between snail and soil samples (Fig. 7). Beta diversity is a measure of species variation between samples. The snail and soil microbiota showed key differences at the phylum level with snail samples dominated by Proteobacteria, while soil samples were dominated by Actinobacteria. The proportion of taxonomically unassigned ASVs in snail and soil microbiota samples was around 20% and 30%, respectively.

## Discussion

Parasitic diseases are a burden on agricultural output globally. In the UK it is estimated that fasciolosis causes £40 million per annum in losses to the economy, primarily due to liver condemnation [27]. Recent research on other parasitic diseases has demonstrated the importance of the roles played by bacterial symbionts. For example, recent malaria research has highlighted the importance of the microbiota of insect vectors for parasite transmission, with certain constituents of the microbiome capable of inhibiting parasite development [28, 29]. However, research on the roles played by the microbiota of helminth intermediate hosts is scarce. In the study reported here, we profiled the microbiome associated with *G. truncatula*, the intermediate host for many flukes in Northern Ireland, by analysing various diversity metrics, including alpha and beta diversity.

The samples collected from each of the two farms included in the study showed no significant differences in alpha diversity (Fig. 1), but we did observe significant differences in the beta diversity metrics between farms (Fig. 2). We have shown field-collected snails may be associated with a diverse microbiota, which can be distinct to the external microbiota, indicating that during the procedures from sampling to sequencing, no exogenous DNA was introduced into the samples. We noted that there was an obvious segregation of clusters within ellipses, which we resolved by stratifying both farm and infection status (Additional file 7: Figure S7). We observed three clusters, indicating that infection status remained a significant factor regardless of farm; the fourth cluster for infected snails from Farm 2 was not observed, likely due to the low sample number (*n* = 2) for this group. Overall, the soil microbiota showed greater bacterial diversity than the snail samples when alpha diversity metrics, such as the Simpson, Shannon and Fisher indexes, were applied. However, although soil diversity is receiving more attention in studies [30–32], it remains a very complex area, with numerous studies showing a relatively large proportion of unassigned/unclassified reads. This issue was reflected in our study with samples from both snail and soil microbiota containing 20% and 30% of taxonomically unassigned ASVs, respectively (Additional file 7: Figure S7; Additional file 8: Table S1; Additional file 9: Table S2; Additional file 10: Table S10).

Research in other snail species has highlighted the presence of a core microbiome that is highly adapted to its host. A transient microbiome is in a state of flux and depends on the environment [33]. Huot et al. characterised the core microbiome in *Biomphalaria* and reported that Proteobacteria is a key component of the core microbiome [34]. This genus was also identified in *Lymnaea stagnalis*, which is closely related to *G. truncatula *[35]. Based on the high number of ASVs assigned to Proteobacteria and Tenericutes, it is likely that these bacteria are a component of the core microbiome in *G. truncatula* as they have been linked to lactic acid production (*Enterobacterales*), which aids in the digestion of cellulose and fermentation of food (*Flavobacter*) [36, 37]. A core microbiome has also been suggested by the results of older studies on different mollusc species [38, 39] and was observed in the data of the present study, indicating that certain phyla are a common component of the core microbiome of the invertebrate hosts of trematodes. Additionally, the shell of the snail, which was removed for the present study, could be an interesting material for the study of transient microbiota or organisms which inhabit the shell (i.e. rotifers). The core and transient microbiomes of the intermediate hosts of trematodes are an interesting subjects for future research.

Geographically distinct populations of snails and potential differences in abiotic factors may contribute to characteristic microbial populations in their composite microbiome. Abiotic factors, such as pH, temperature and proximity to water, will play a crucial role determining which microorganisms will be able to grow [30]. These factors may be different between the farms from which the snails were collected, thus influencing their respective microbiomes. Additional abiotic factors, such as microplastic pollution, have been shown to impact the microbiota in *Lymnaea stagnalis*, which is a closely related species to *G. truncatula* [35]*.* These factors should be considered further in future studies to determine the impact these microbiota present to invertebrates.

Variation in the susceptibility of *Biomphalaria* snails to schistosomes has been recognised since 1949 [40]. Recent studies have highlighted that host genetics are partially responsible [41]; not only have specific alleles been associated with a resistant phenotype in *Biomphalaria,* but also the snail-associated microbiota in susceptible populations has been found to differ from the one identified in resistant populations [42]. Another study highlighted that specific genotypes involved in snail susceptibility to infection correlated to changes in the abundance of *Gemmatimonas aurantiaca* and *Micavibrio aeruginosavorus* [19]*.* However, it is important to note that these genes likely play a role in the regulation of all microbiota within the host and may not be specifically targeting these species. While snail genetics certainly plays a role in the susceptibility to infection, genetics is likely just another player in a complex interaction between snail, parasite and microbiota. Further studies are needed to functionally characterise probiotic bacteria and investigate genera associated with specific genotypes underlying snail susceptibility to infection.

These data indicate that the microbiome of field collected snails in Northern Ireland is rich and diverse, which is consistent with the findings of other studies on mollusks, crustaceans and insects [43–47]. We also observed differences in the microbiota between naïve and infected snails, irrespective of the farm from which the snails were collected. Although we did not explore the functional or mechanistic roles with our 16S rRNA data, the known functions of certain genera present in our data can be useful to infer possible processes. We highlight that there are genera that are more prevalent depending on whether the snail is infected with a trematode (Fig. 5; Table 1). Of these genera, we found that *Mycoplasma, Bacteroides* and *Planctomycetes* are more prevalent in infected snails. *Mycoplasma* is an obligate pathogen in vertebrates; however, the role it plays in snails is unknown. We note an increase in the prevalence of *Bacteroides* in infected snails; these bacteria inhabit the gastrointestinal tract of animals [48] and while they can be opportunistic pathogens [49], they are also known to be probiotic to their hosts and to release molecules stimulating the host immune system [48]. The increase in the prevalence of *Bacteroides* could be in response to the stress of parasitism on the host. *Planctomycetes* has been previously reported in freshwater snails; however the role it plays remains unknown [50]. In uninfected snails we observed an increase in *Firmicutes,* which is known to play a role in the absorption of nutrients [51]. The differences between infected and naïve snails may arise for several reasons. The stress of parasitism on the host immune system may be a significant driver of microbial change [11]; for example, opportunistic pathogens may take advantage of the stress on the host immune system caused by parasitic infection. Another possible explanation for these changes is the secretion of antimicrobial peptides in extracellular vesicles [52]; such secreted peptides by the parasite could serve to reduce the number of commensal microorganisms in the snail microbiome. Recent research by Bowden et al. [53] has indicated that the haemolymph extracellular vesicles in a marine mollusc contain a fibrinogen domain containing protein that plays a key role in immune defence; similar proteins have been found to play a role in the freshwater snail *Biomphalaria glabrata* and schistosome infections [54]. Furthermore, infective miracidia may potentially introduce additional microorganisms that facilitate the establishment of a parasitic infection [19].

The invertebrate host microbiota has been shown to play a protective role in malaria transmission; however, this interaction is not unique to the malaria model. Similar observations have been reported between the sandfly microbiome and infection by *Leishmania *[55], in tsetse flies during the transmission of trypanosomiasis [56] and in the freshwater *Biomphalaria* snails and Schistosoma *mansoni*, [19]. These interactions may play a role in infection outcomes and potentially in the evolution of host parasite systems [57, 58]. *Biomphalaria* and *Galba* snails both belong to the superorder Hygrophila, so there are likely similarities in their immune responses to parasitic infection. A recent study has shown that the haemolymph of *Biomphalaria* has a microbiome [19], and it is known that the haemolymph plays a key role in the snail immune response to the invading schistosome through both humoral factors and haemocytes [59]. The microbiome associated with the invading miracidia could also play a role in these interactions; however there is insufficient research on the role of parasite microbiomes [60] [61]. This deficiency is addressed by the Parasite Microbiome Project [62], highlighting the tentative roles microbes play in host systems by facilitating host switching or inducing genetic changes in host microbiota via novel microbe-microbe interactions. The microbiome could aid the snail response to infection either through direct interaction with the parasite or by augmenting the snail immune response; however, further research is needed to define these roles.

The findings reported here suggest the presence of a snail-associated core microbiota that differs from microbe communities dwelling in the shared environment with the snail. Our results warrant further validation through controlled laboratory experiments that overcome the limitations of this field study. One of these limitations was the inability to calculate sample size, and thus estimate statistical power. Indeed, since the present investigation is, to the best of our knowledge, the first of its kind, calculations of effect size—and thus of sample size—is challenging. On the other hand, this is a pilot study that will guide the estimation of these parameters in future field investigations. Under experimental controlled conditions, the effect of trematode infections on the snail microbiota can be better assessed [11]. Furthermore, while we only determined infection with trematode parasites, there are other pathogens of snails which could tentatively influence the microbiota present in the snails, such as protozoans, viruses or other bacteria [63]. Additionally, other confounding variables, such as snail age and size, are unknown in this study. It has been suggested that climate could also play a role in influencing changes in microbiota [64].

## Conclusion

In conclusion, we have demonstrated that *G. truncatula,* the intermediate host of many trematode parasites, harbours an internal microbiome different to that of the soil environment it inhabits. In addition, we have shown that the microbiome composition at the bacterial genus level of trematode-infected snails differs from that of naïve snails. There are many potential drivers for these changes, including the stress induced by parasitism, the secretion of antimicrobial peptides by either the parasite or the host and/or the presence of opportunistic pathogens in the snail microbiota. Further work is required to determine the role of these microorganisms within the snail associated with metabolism, immunity, parasite development and thus transmission.

### Supplementary Information

**Additional file 1: Figure S1.** Images taken at the collection site for farm 1.

**Additional file 2: Figure S2.** Images taken at the collection site for farm 2.

**Additional file 3: Figure S3.** A gel electropherogram of PCR products to test for infection. 1% agarose gel with SYBR added. A 100 bp ladder was used with an approximate product size of 500 bp. Double bands shown represent coinfection. DEPC treated water was added as a negative control while *Fasciola hepatica* and *Calicophoron daubneyi* were used respectively as positive controls.

**Additional file 4: Figure S4.** Cumulative reads counts for all samples presented at the Phylum level stratified by farm and sample type.

**Additional file 5: Figure S5.** Alpha rarefaction curves for all samples, each represented by a different colour. Samples 28Jan22-EXT03 and 28Jan22-EXT09 were excluded from the analysis based on low read depth observed in the alpha rarefaction.

**Additional file 6: Figure S6.** A summary of library size for each sample. Points in red are controls, green are samples collected from farm 1 and blue are samples collected from farm 2.

**Additional file 7: Figure S7.** Beta diversity of *Galba truncatula* snails collected from each farm and their infection status (U: Uninfected, I: Infected), presented at the Genus level. **A** Beta diversity compared using Bray–Curtis dissimilarity (PCoA) distances revealing significant differences in the diversity of the microbiota of snails collected from each farm including infection status, [PERMANOVA] F-value: 3.96; R-squared: 0.34; p-value: 0.001. **B** Beta diversity compared using Jensen-Shannon divergence (PCoA) distances revealing significant differences in the diversity of the microbiota of snails collected from each farm including infection status, [PERMANOVA] F-value: 7.21; R-squared: 0.48; p-value: 0.001. **C** Beta diversity compared using Jaccard index (PCoA) distances revealing significant differences in the diversity of the microbiota of snails collected from each farm including infection status, [PERMANOVA] F-value: 3.06; R-squared: 0.29; p-value: 0.001.

**Additional file 8: Table S1.** Summary of sample metadata used in all analyses.

**Additional file 9: Table S2.** Resulting ASV count table with integrated classification results from QIIME2.

**Additional file 10: Table S3.** The results from LEfSe analysis stratified (tabs) from Phylum to Genera.

## Data Availability

The dataset supporting the conclusions of this article is available in the NCBI Sequencing Read Archive repository. PRJNA992680 – https://www.ncbi.nlm.nih.gov/sra/?term=PRJNA992680
