# Supplementary figures and images for: Assessing the microbiota of the snail intermediate host of trematodes, Galba truncatula
Source: Parasit Vectors. 2024 Jan 23;17:31. doi: 10.1186/s13071-024-06118-7 (PMC10807216; doi:10.1186/s13071-024-06118-7)

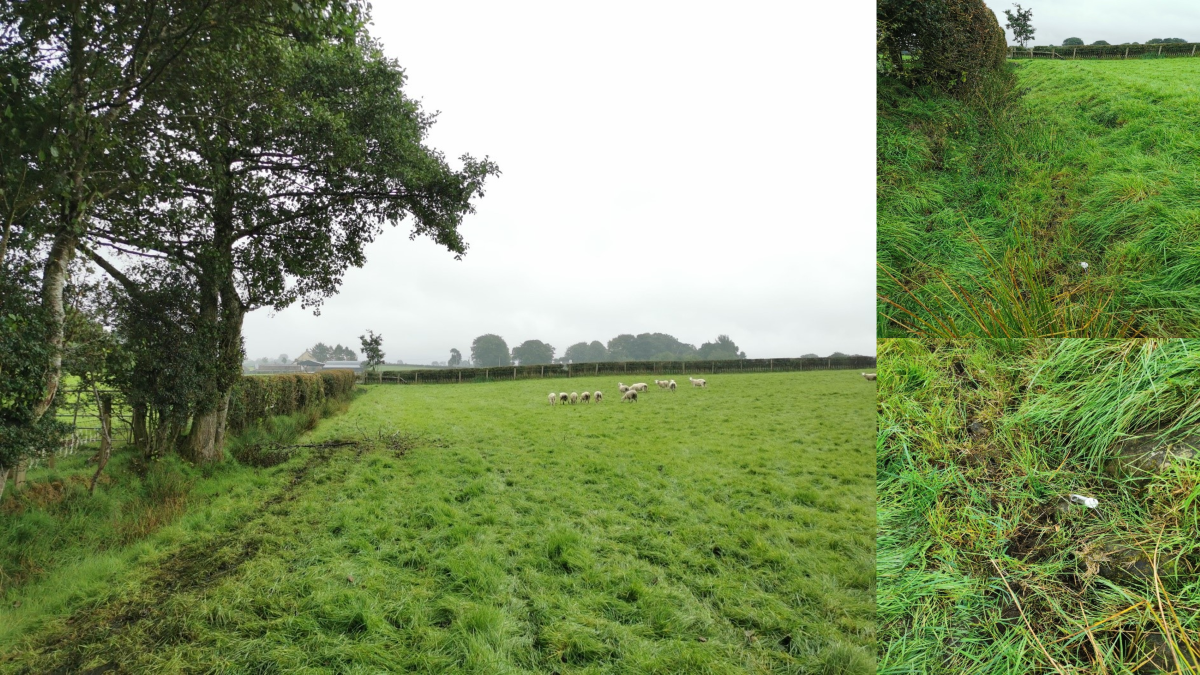

Supplement: Supplementary file 1 — Additional file 1: Figure S1. Images taken at the collection site for farm 1. [file 13071_2024_6118_MOESM1_ESM.png]

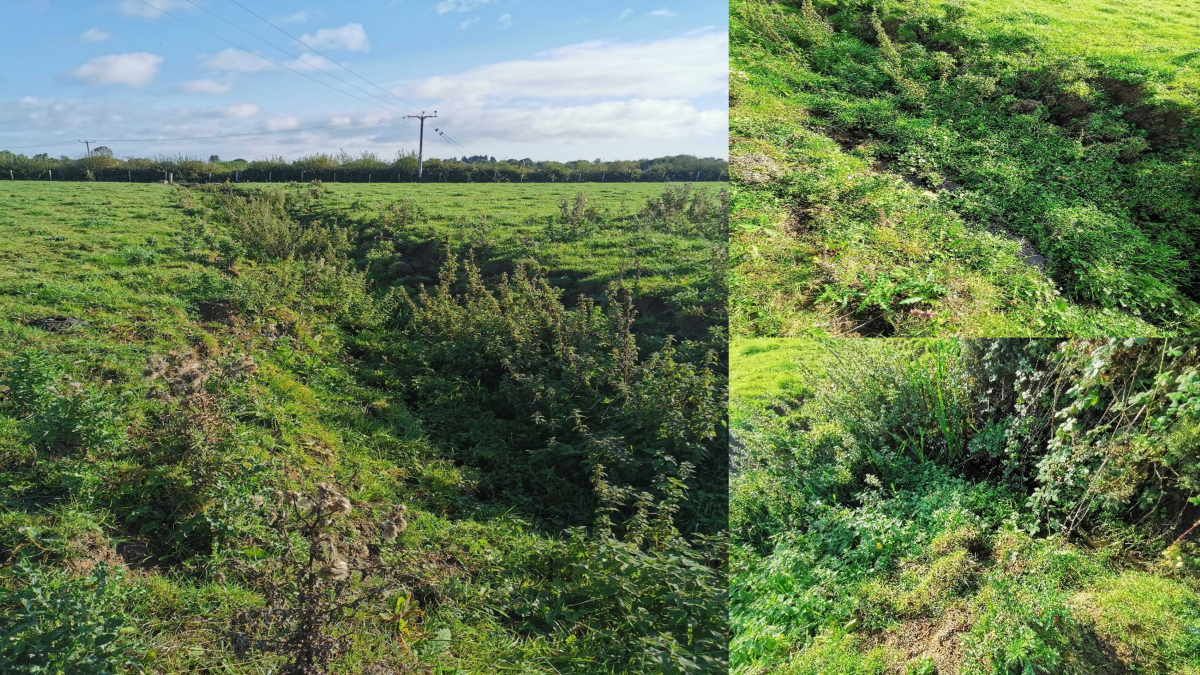

Supplement: Supplementary file 2 — Additional file 2: Figure S2. Images taken at the collection site for farm 2. [file 13071_2024_6118_MOESM2_ESM.png]

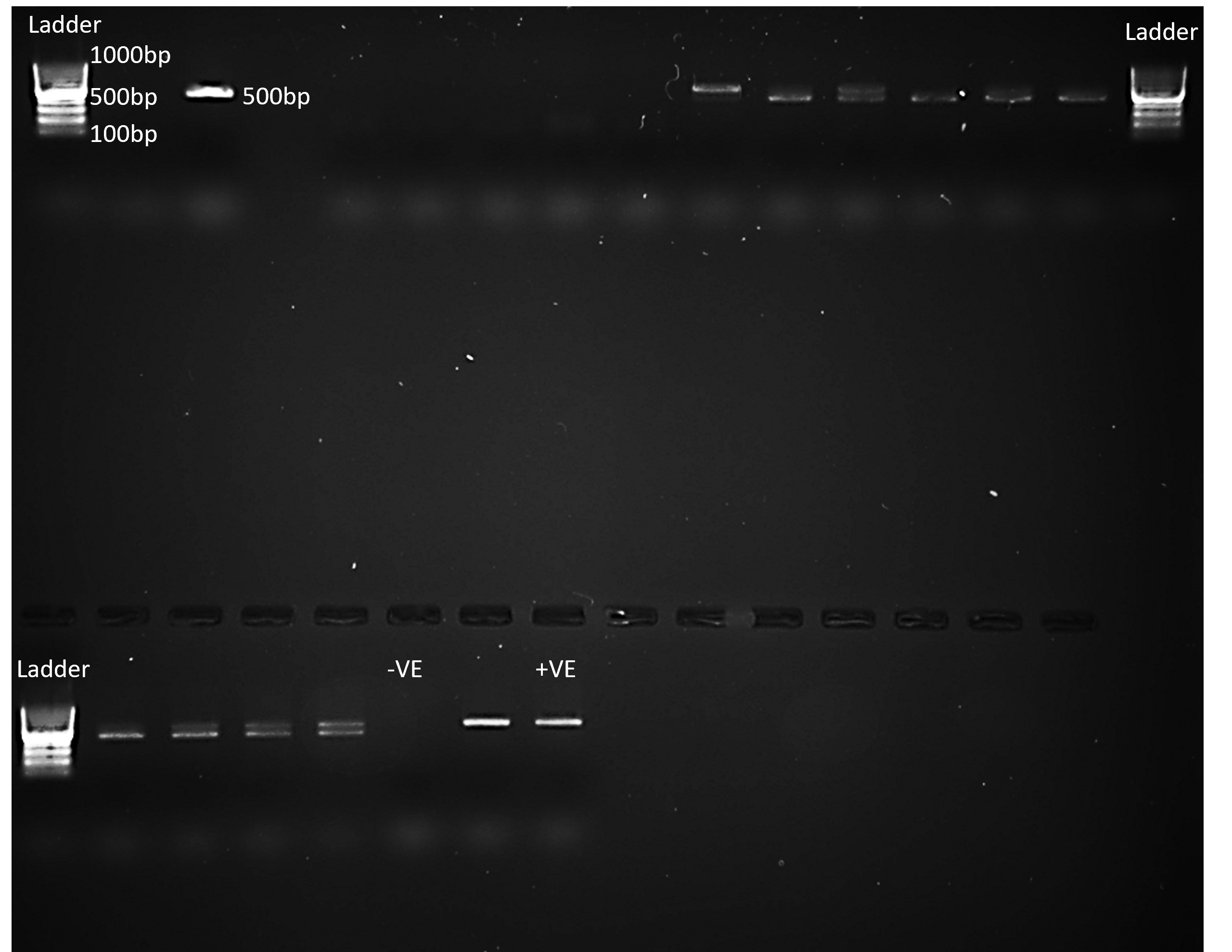

Supplement: Supplementary file 3 — Additional file 3: Figure S3. A gel electropherogram of PCR products to test for infection. 1% agarose gel with SYBR added. A 100 bp ladder was used with an approximate product size of 500 bp. Double bands shown represent coinfection. DEPC treated water was added as a negative control while Fasciola hepatica and Calicophoron daubneyi were used respectively as positive controls. [file 13071_2024_6118_MOESM3_ESM.png]

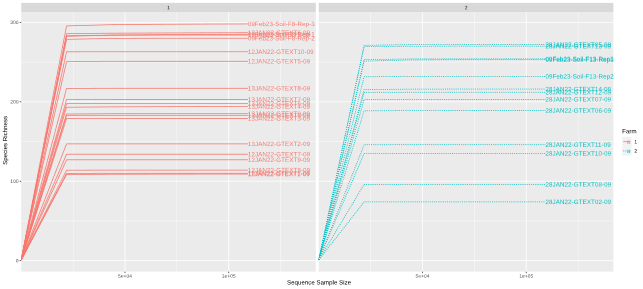

Supplement: Supplementary file 5 — Additional file 5: Figure S5. Alpha rarefaction curves for all samples, each represented by a different colour. Samples 28Jan22-EXT03 and 28Jan22-EXT09 were excluded from the analysis based on low read depth observed in the alpha rarefaction. [file 13071_2024_6118_MOESM5_ESM.png]

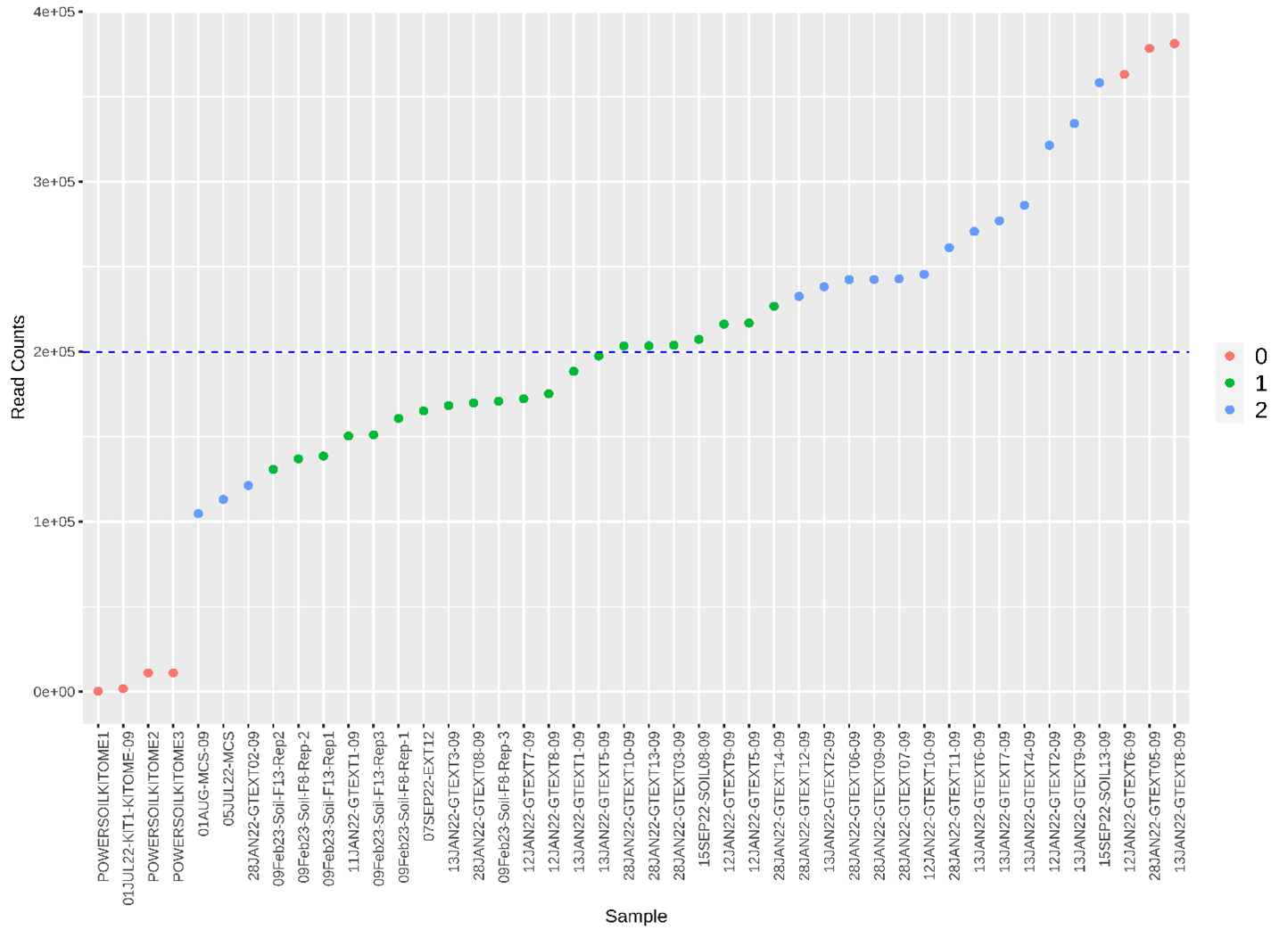

Supplement: Supplementary file 6 — Additional file 6: Figure S6. A summary of library size for each sample. Points in red are controls, green are samples collected from farm 1 and blue are samples collected from farm 2. [file 13071_2024_6118_MOESM6_ESM.png]
